# Short-Time Impedance Spectroscopy Using a Mode-Switching Nonsinusoidal Oscillator: Applicability to Biological Tissues and Continuous Measurement

**DOI:** 10.3390/s21216951

**Published:** 2021-10-20

**Authors:** Tomiharu Yamaguchi, Emiyu Ogawa, Akinori Ueno

**Affiliations:** 1 Department of Electrical and Electronic Engineering, Tokyo Denki University, Tokyo 120-8551, Japan; 21769@ms.dendai.ac.jp; 2 School of Allied Health Science, Kitasato University, Kanagawa 252-0373, Japan; e.ogawa@kitasato-u.ac.jp

**Keywords:** capacitive coupling, impedance spectroscopy, nonsinusoidal oscillator, DFT, frequency switching, biological application, continuous impedance measurement

## Abstract

Herein, we propose an impedance spectroscopy method using a mode-switching nonsinusoidal oscillator and apply this method for measuring the impedance of biological tissues and continuous impedance measurement. To obtain impedance spectra over a wide frequency range, we fabricated a novel nonsinusoidal oscillator incorporating binary counters and analog switches. This oscillator could periodically switch oscillation frequency through the mode switching of the feedback resistor. From the oscillation waveform at each oscillation frequency of this circuit (oscillator), we determined the impedance spectrum of a measured object using the discrete-time Fourier transform. Subsequently, we obtained the broad impedance spectrum of the measured object by merging odd-order harmonic spectral components up to the 19th order for each oscillation frequency. From the measured spectrum, the resistive and capacitive components of the circuit simulating bioimpedance were estimated with high accuracy. Moreover, the proposed method was used to measure the impedance of porcine myocardium; changes in the impedance spectrum of the myocardial tissue due to coagulation could be measured. Furthermore, rapid variations in the resistance value of a CdS photocell could be continuously measured using the proposed method.

## 1. Introduction

Bioelectrical impedance analysis has become an important technique in the medical and healthcare fields [1,2,3,4,5,6,7,8,9]. Because the impedance of a living body (bioimpedance) depends on body composition, such as the water content of the body, it reflects medical conditions [10,11,12,13]. Thus, the absolute value and phase of bioimpedance are used to diagnose diseases, and this process is known as bioelectric impedance vector analysis [14,15,16,17,18,19]. Impedance measurement is also used in catheter ablation for treating arrhythmia [20,21,22,23]. By measuring the local impedance at an ablation site with electrodes integrated into an ablation catheter, the cauterization of myocardial tissue can be monitored. In the medical field, impedance may be measured using a single frequency or a few frequencies. However, bioimpedance has frequency characteristics over a wide range of frequencies, which originate from impedance components such as intracellular fluid resistance, extracellular fluid resistance, and the capacitance of cell membranes. Because cell membranes are highly insulating and cell fluids have ionic conductivity, in a low-frequency range, the current barely flows within cells but flows through the gaps among cells. Conversely, in a high-frequency range, the impedance caused by the capacitance of cell membranes decreases, meaning the current flows uniformly inside and outside cells. Therefore, if the impedance spectrum over a wide frequency range could be measured rapidly and conveniently, more detailed information about biological tissues could be obtained, which would be useful for diagnosing and treating medical conditions.

Impedance measurement generally involves coupling an electrode to a measured object through resistive coupling. Therefore, noninvasive impedance spectroscopy targeting insulated resistors, for instance, a living body covered with insulator-like clothing, has rarely been performed. To address this gap, we previously proposed capacitive coupling impedance spectroscopy (CIS) as a noninvasive technique for measuring impedance spectra through an insulation coating [24]. In this method, the measured object and capacitive coupling part are incorporated into an oscillation circuit. The oscillation frequency of the circuit depends on the coupling capacitance and the impedance of the measured object. Thus, even if the equivalent circuit model of the measured object is unknown, the change in these parameters can be monitored by measuring the oscillation frequency. This is applicable to monitoring a body movement and the breathing effort of a living body [25]. However, the equivalent circuit parameters of the measured object cannot be uniquely determined by only the oscillation frequency.

The frequency spectrum of the complex impedance of the measured object is obtained in a short time by analyzing the obtained oscillation waveform using the discrete-time Fourier transform (DFT), which allows estimating the resistance and capacitance components of the measured object. Therefore, CIS can be applied to not only measurement applications such as ablation by resistively contacting electrodes to biological tissue but also the measurement of biological impedance through an insulator such as clothing. Consequently, it is suitable for fabricating portable/wearable measurement systems. Furthermore, because the impedance spectrum is obtained from the oscillation waveform in every cycle, it enables an almost continuous impedance measurement. Continuous measurement of the impedance spectrum can be used to monitor biological processes such as cell differentiation [26].

In our previous CIS measurement, we used a nonsinusoidal oscillator incorporated into the measured object to estimate the impedance spectrum. DFT analysis of the nonsinusoidal wave obtained from this oscillator can determine the impedance at the fundamental frequency of the waveform and its odd harmonic frequencies. If the coupling capacitance is constant, and the measurement system has a high signal-to-noise ratio, the equivalent circuit parameters can be determined via less frequency points. However, in practical application, the coupling capacitance is often unknown and may fluctuate. To determine the equivalent circuit parameters of the measured object with high accuracy, we must estimate the impedance spectrum in the frequency range where the impedance has high frequency dependence. However, the oscillation frequency range of the nonsinusoidal oscillator is limited by the feedback resistance of it. Therefore, the feedback resistance cannot be set appropriately for an unknown coupling capacitance, and the impedance spectrum in the frequency range required for circuit parameter estimation may not be measured.

To solve this problem, we designed a nonsinusoidal oscillator that can change the oscillation frequency drastically by switching several feedback resistors. Using the significantly different values of the feedback resistances, even if the coupling capacitance fluctuates, one of the feedback resistors can cover the frequency range required for circuit parameter estimation and thus leads to a stable and high estimation accuracy.

Furthermore, high electrical noise may reduce the measurement accuracy of the impedance spectrum. In this measurement environment, it is difficult to estimate the circuit parameters accurately with less frequency points. Our proposed frequency-switchable (mode-switching) oscillator enables impedance measurement with many frequency points by merging the frequency spectra for each feedback resistance.

To apply our proposed nonsinusoidal oscillator to bioimpedance measurement, this study aimed to demonstrate the following four outcomes using CIS:

(1) Design a nonsinusoidal oscillation circuit capable of periodically switching oscillation frequency;
(2) Show that the proposed method can estimate circuit parameters for circuit models close to actual measured objects;
(3) Apply the proposed method to the impedance measurement of biological tissues;
(4) Show that the continuous measurement of impedance is possible.

## 2. Capacitive Coupling Impedance Spectroscopy

### 2.1. Mode-Switching Nonsinusoidal Oscillator

In CIS, a nonsinusoidal oscillator circuit, such as a Schmitt trigger inverter oscillator, is used. Although this oscillation circuit does not have a high-accuracy oscillation frequency, it allows constructing a system that is small, inexpensive, and easy to handle because it has few parts, and the oscillation is stable. In our previous CIS study, the impedance of the measured object at the fundamental frequency (oscillation frequency) and odd-order harmonics were determined using DFT analysis of the oscillation waveforms. However, in some cases, the impedance spectra of sufficient data points cannot be obtained with a single oscillation frequency, and the estimation accuracy decreases when the circuit parameters are estimated using an equivalent circuit model. To address this problem, we designed a novel mode-switching nonsinusoidal oscillator that can switch among multiple oscillating frequencies.

**Figure 1:**
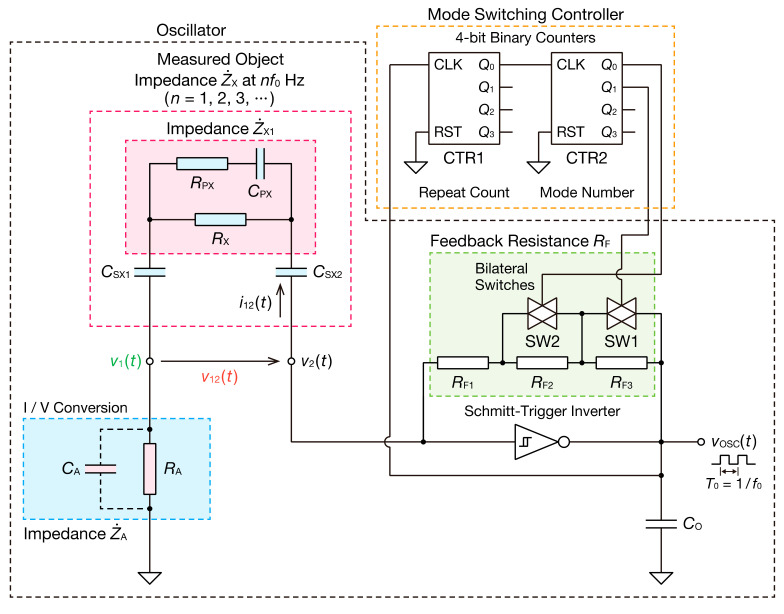
Mode-switching nonsinusoidal oscillator. The oscillation frequency $f_{0}$ and the oscillation period $T_{0}$ of the oscillator are switched between four different frequencies by 4-bit binary counters and bilateral analog switches. CLK of 4-bit binary counters denotes a clock signal, and RST represents a reset signal. $Q_{0}$, $Q_{1}$, $Q_{2,}$ and $Q_{3}$ of 4-bit binary counters denote zero bits, and the first, second, and third bits of the output signals, respectively. $C_{O}$ represents a bypass capacitor to remove the switching noise in the oscillator, and its value is 0.47 nF. CTR1 and CTR2 are binary counters to control the repeat count and the mode number of the mode switching, respectively.

Figure 1 shows a schematic of the mode-switching nonsinusoidal oscillator. A nonsinusoidal oscillation waveform corresponding to the impedance of the measured object is obtained by incorporating the measured object, capacitively coupled with the electrode, into the oscillation circuit. ${\dot{Z}}_{X}$ represents the impedance of the measured object, including capacitive coupling, whose circuit parameters differ depending on the equivalent circuit model of the measured object. For example, in the case of bioimpedance, it comprises extracellular fluid resistance $R_{X}$, intracellular fluid resistance $R_{\mathrm{PX}}$, and the capacitance of cell membranes $C_{\mathrm{PX}}$ [27,28]. In particular, $R_{\mathrm{PX}}=$ 0 Ω for skin impedance [29]. ${\dot{Z}}_{X1}$ is the impedance of the measured object excluding capacitive coupling. Capacitances $C_{\mathrm{SX}1}$ and $C_{\mathrm{SX}2}$ correspond to the coupling capacitance between the insulator and electrode. ${\dot{Z}}_{A}$ is the combined impedance of resistance $R_{A}$ and parasitic capacitance $C_{A}$. The current $i_{12}\left(t\right)$ flowing into ${\dot{Z}}_{X}$ is calculated from the voltage $v_{1}\left(t\right)$ generated in ${\dot{Z}}_{A}$ (I/V conversion). The resistance $R_{A}$ represents the resistance for measuring the current. $C_{A}$ represents a parasitic component caused by the parasitic capacitance of the circuit board, input capacitance of the probe of the oscilloscope, and other factors. The feedback resistance $R_{F}$ of the oscillator circuit comprises resistors $R_{F1}$, $R_{F2},$ and $R_{F3}$ connected in series. The Schmitt trigger inverter switches the high and low levels of the voltage $v_{\mathrm{OSC}}\left(t\right)$, $V_{H}$ and $V_{L}$, by the input voltage $v_{2}\left(t\right)$. The voltage $v_{2}\left(t\right)$ changes by the charge/discharge of the capacitors connected on the input side of the Schmitt trigger inverter, which causes the square wave of the voltage $v_{\mathrm{OSC}}\left(t\right)$. In addition, square-like and triangle-like waveforms are generated as $v_{1}\left(t\right)$ and $v_{2}\left(t\right)$, respectively. Because the charging/discharging speed of the capacitors is determined by the current $i_{12}\left(t\right)$ flowing through ${\dot{Z}}_{X}$, the oscillation frequency depends on ${\dot{Z}}_{X}$, ${\dot{Z}}_{A}$, and $R_{F}$. If ${\dot{Z}}_{X}$ is a resistive element $R_{X}$ and capacitive coupling $C_{\mathrm{SX}}$ in series, and parasitic capacitance $C_{A}$ can be ignored, the oscillation frequency $f_{0}$ can be expressed with the following [24]:

(1) $$f_{0}=\frac{1}{\left(R_{F}+R_{A}+R_{X}\right)C_{\mathrm{SX}}\left(\ln\frac{V_{H}-V_{A}}{V_{H}-V_{P}}+\ln\frac{V_{B}-V_{L}}{V_{N}-V_{L}}\right)},$$

(2) $$V_{A}=V_{N}+\frac{R_{A}+R_{X}}{R_{F}+R_{A}+R_{X}}\left(V_{H}-V_{L}\right),$$

(3) $$V_{B}=V_{P}-\frac{R_{A}+R_{X}}{R_{F}+R_{A}+R_{X}}\left(V_{H}-V_{L}\right),$$

where $C_{\mathrm{SX}}=C_{\mathrm{SX}1}C_{\mathrm{SX}2}/\left(C_{\mathrm{SX}1}+C_{\mathrm{SX}2}\right)$ denotes the combined capacitance of $C_{\mathrm{SX}1}$ and $C_{\mathrm{SX}2}$. $V_{P}$ and $V_{N}$ are the threshold voltages of the Schmitt trigger inverter. If ${\dot{Z}}_{X}$ has a more complex circuit network, these equations are not strictly valid, but the oscillation frequency can be roughly estimated using these equations.

Because the operation of the bilateral switches can cause an unstable oscillation such as switching noise, we used the 4-bit binary counter CTR1 to stabilize the oscillation. The counter CTR1 controls the repeat count for switching the feedback resistors. The square wave signal $v_{\mathrm{OSC}}\left(t\right)$ of the oscillator is input to the counter CTR1 as the clock signal. The 0-bit output $Q_{0}$ of the counter CTR1 has half the frequency of $v_{\mathrm{OSC}}\left(t\right)$. For this reason, the bilateral switches operate every two cycles of the oscillation waveform for each $R_{F}$, and the oscillation of the second half cycle becomes stable.

Subsequently, the frequency-divided square wave signal is input as a clock signal to the counter CTR2. CTR2 determines the mode number obtained from the mode switching of the oscillator. Moreover, the 0-bit and 1-bit outputs $Q_{0}$ and $Q_{1}\;$ of the counter CTR2 are input as the control signals of the bilateral switches SW1 and SW2, respectively. Because $R_{F2}\;$ or $R_{F3}\;$ is short circuited when the switch is ON, $R_{F}$ is switched every two cycles of the oscillation waveform in four modes: $R_{F1}$, $R_{F1}+R_{F2}$, $R_{F1}+R_{F3}$, and $R_{F1}+R_{F2}+R_{F3}$ (Table 1). Therefore, the oscillation frequency can be switched periodically.

The procedure for estimating impedance is determined using a previously reported procedure [24]. The voltages $v_{1}\left(t\right)\;$ and $v_{12}\left(t\right)\;$ measured in the circuit, shown in Figure 1, form the periodic nonsinusoidal waveforms of the oscillation frequency $f_{0}$. Therefore, the Fourier series expansion of the one cycle of the observed waveform comprises a sine wave of the fundamental frequency $f_{0}$ and its integer multiple $kf_{0}$ (k=1, 2, 3, ⋯), and the coefficients of each frequency component are obtained as the amplitude and phase spectra through the DFT of the section concerned. From the obtained amplitude and phase spectra, ${\dot{Z}}_{X}\;$ can be calculated using the following equation:

(4) $${\dot{Z}}_{X}\left(kf_{0}\right)=\frac{{\dot{V}}_{12}\left(kf_{0}\right)}{{\dot{I}}_{12}\left(kf_{0}\right)}=\frac{\left|{\dot{V}}_{12}\left(kf_{0}\right)\right|}{\left|{\dot{V}}_{1}\left(kf_{0}\right)\right|}\cdot \left|{\dot{Z}}_{A}\left(kf_{0}\right)\right|e^{j\left\{\theta_{V12}\left(kf_{0}\right)-\theta_{V1}\left(kf_{0}\right)+\theta_{\mathrm{ZA}}\left(kf_{0}\right)\right\}},$$

where ${\dot{V}}_{12}\left(kf_{0}\right)$ and ${\dot{V}}_{1}\left(kf_{0}\right)$ denote the complex voltages of $v_{12}\left(t\right)$ and $v_{1}\left(t\right)$, respectively; ${\dot{I}}_{12}\left(kf_{0}\right)$ denotes the complex current of $i_{12}\left(t\right)$; $\left|{\dot{V}}_{12}\left(kf_{0}\right)\right|$ and $\theta_{V12}\left(kf_{0}\right)$ represent the amplitude and phase of the complex voltage at ${\dot{Z}}_{X}$; $\left|{\dot{V}}_{1}\left(kf_{0}\right)\right|\;$ and $\theta_{V1}\left(kf_{0}\right)$ represent the amplitude and phase of the complex voltage at ${\dot{Z}}_{A}$; and $\left|{\dot{Z}}_{A}\left(kf_{0}\right)\right|$ and $\theta_{\mathrm{ZA}}\left(kf_{0}\right)$ represent the magnitude and phase of the impedance ${\dot{Z}}_{A}$, respectively. The impedance estimation using Equation (4) is valid for any periodic waveform of $v_{12}\left(t\right)$ and $v_{1}\left(t\right)$ because Equation (4) is applied to each frequency component obtained from the oscillation waveform of $v_{12}\left(t\right)$ and $v_{1}\left(t\right)$. The real and imaginary parts of the impedance ${\dot{Z}}_{X}$, ReZ˙X and ImZ˙X, are obtained with Equations (5) and (6):

(5) ReZ˙X=V˙12nf0V˙1nf0·Z˙Anf0cosθV12nf0−θV1nf0+θZAnf0,

(6) ImZ˙X=V˙12nf0V˙1nf0·Z˙Anf0sinθV12nf0−θV1nf0+θZAnf0.

We can obtain the oscillation waveform with multiple oscillation frequencies using the mode-switching oscillator. Therefore, one synthesized impedance spectrum is obtained by merging the spectral data for each oscillation frequency.

If it is known that ${\dot{Z}}_{X}$ is a series connection of the resistor $R_{X}$ and the capacitive coupling $C_{\mathrm{SX}}$, $R_{X}$ and $C_{\mathrm{SX}}$ can be estimated using the least squares method for a finite number of frequency spectra $nf_{0}$ (n=1, 2, 3, ⋯, N).

(7) ReZ˙X=RX,

(8) ImZ˙X=−12πnf0CSX.

Furthermore, when ${\dot{Z}}_{X}$ is the common bioimpedance (Figure 1) equivalent circuit model,

(9) ReZ˙X=2πnf0CPX2RXRPX2+RX2RPX+RX2πnf0CPX2RPX+RX2+1,

(10) ImZ˙X=−12πnf0CSX−2πnf0CPXRX22πnf0CPX2RPX+RX2+1.

Similarly, the circuit parameters can be estimated using the least squares method. When $R_{\mathrm{PX}}=$ 0 Ω, Equations (9) and (10) represent the real and imaginary parts of the circuit comprising the parallel-connected $R_{X}$ and $C_{\mathrm{PX}}$ (RC parallel circuit) and coupling capacitance $C_{\mathrm{SX}}$, respectively.

### 2.2. Experimental Method

#### 2.2.1. CIS of Parallel RC Circuits and Bioimpedance Models

A readily available 74HC14AP (Toshiba, Japan) was used as the Schmitt trigger inverter integrated circuit (IC), as illustrated in Figure 1. The typical $V_{P}$ and $V_{N}$ values of 74HC14AP in Equations (1)–(3) are 3.5 V and 2.3 V at 6 V of the supply voltage, respectively. A TC4520BP (Toshiba, Japan) was used as the 4-bit binary counter IC, and a TC74HC4066AP (Toshiba, Japan) was used as the bilateral analog switch IC. The power supply voltage of these ICs was set to 6 V, and $V_{H}$ and $V_{L}$ were set to 6 V and 0 V, respectively. ${\dot{Z}}_{A}$ was considered to consist only of the resistor $R_{A}$. $R_{A}$ was set to 0.20 Ω. There is typically $C_{A}$ of several tens of pF due to the parasitic components such as the probe capacity of the oscilloscope, but $C_{A}$ is negligible because $R_{A}$ is sufficiently small. The oscillator was designed to measure the impedance of biological tissue with a resistance of less than 1 kΩ and a capacitance of several nF. We selected four fundamental frequencies so that they contribute to an accurate estimation of the measured impedance parameters based on preliminary experiments. Two sets of the fundamental frequencies obtained by combining $R_{F1}$, $R_{F2}$, and $R_{F3}$ are shown in Table 2.

First, circuit parameters comprising the RC parallel circuit ($R_{X}$ and $C_{\mathrm{PX}}$) and coupling capacitance $C_{\mathrm{SX}}$ were estimated using CIS. Metal film resistors and polypropylene (PP) film capacitors with known resistance and capacitance values were incorporated into the circuit as resistance $R_{X}$ and capacitance $C_{\mathrm{PX}}$, $C_{\mathrm{SX}}$. To demonstrate that our proposed method can estimate $C_{\mathrm{PX}}$ with various values, we set the capacitance of 1.016 or 9.85 nF as $C_{\mathrm{PX}}$. The coupling capacitance $C_{\mathrm{SX}}$ was set to 9.76 nF. We used the combination of set 1 in Table 2 as $R_{F1}$, $R_{F2}$, and $R_{F3}$. These feedback resistors enable determining the impedance spectrum in a wider frequency range.

Further, we estimated the circuit parameters of the bioimpedance model ($R_{X}$, $R_{\mathrm{PX}}$, $C_{\mathrm{PX}}$, and $C_{\mathrm{SX}}$) with the coupling capacitance using CIS. The estimation of $R_{\mathrm{PX}}$ is more difficult than that of $R_{X}$ because it is affected by the accuracy of the impedance estimated using higher-order harmonics. For this reason, we evaluated the estimation accuracy of $R_{\mathrm{PX}}$ in more detail. Resistance with a constant value of 0.30 kΩ was used as $R_{X}$. $C_{\mathrm{PX}}$ was set to 3.22 nF, which simulates the capacitance with biological tissue. $C_{\mathrm{SX}}$ was set to 9.76 nF. To obtain many frequency points at lower frequencies, we used the combination of set 2 in Table 2 as $R_{F1}$, $R_{F2}$, and $R_{F3}$.

In these experiments, we prepared ten resistors as $R_{X}$ or $R_{\mathrm{PX}}$ with different resistances from 0.10 to 1.0 kΩ and set the coupling capacitance $C_{\mathrm{SX}}$ to 9.76 nF.

#### 2.2.2. Application of CIS to Biological Tissues

**Figure 2:**
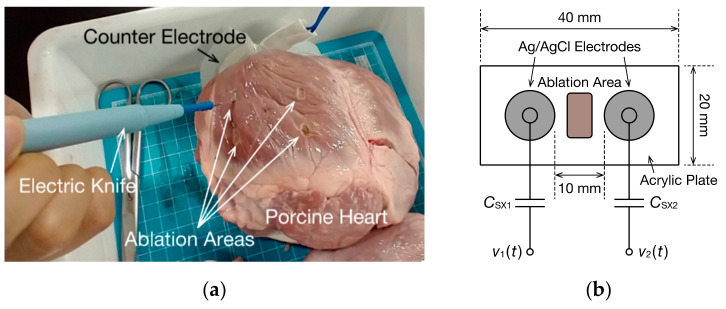
Impedance measurement of porcine myocardium: (**a**) photograph of the porcine left ventricle coagulated using electrocautery; (**b**) measurement electrode fixture.

To demonstrate that the impedance spectrum of biological tissues can be measured using this CIS system, we measured the impedance of porcine myocardium. The coagulation of the myocardium causes tissue degeneration and decreases the impedance of the coagulated part [30]. Therefore, porcine myocardium samples were coagulated through electrocautery to determine whether the change in impedance before and after the coagulation could be measured. Three porcine hearts were prepared, and the myocardium of each left ventricle was ablated in four locations (Figure 2a). Counter electrodes were placed across the back of the porcine myocardium, and it was coagulated through electrocautery for 30 s at a power of 30 W. The electrocautery was set to the soft coagulation mode. Moreover, measuring electrodes were set up, as illustrated in Figure 2b, with circular electrodes on either side of the ablation point. The electrodes were spaced 10 mm apart and directly contacted at the same position of the myocardium before and after each coagulation. The impedance generated when the electrode and myocardial tissue come into contact forms a resistive coupling. Therefore, the coupling capacitance is extremely small, meaning the oscillating frequency of the mode-switching nonsinusoidal oscillator increases. A PP film capacitor of $C_{\mathrm{SX}}=$ 9.76 nF was connected in series between the electrode and oscillation circuit so that the oscillation frequency was reduced to approximately 20 kHz. $R_{F1}$, $R_{F2}$, and $R_{F3}$ were 3.3, 1.8, and 3.0 kΩ (set 1), respectively.

#### 2.2.3. Continuous Impedance Measurement Using CIS

**Figure 3:**
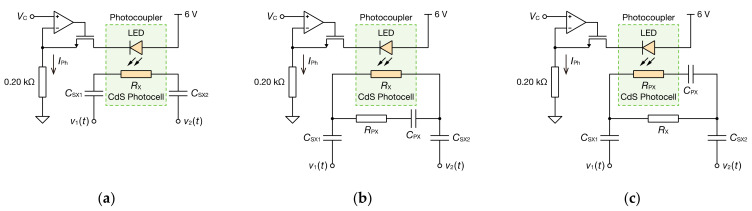
Constant current circuit for the LED current control of the photocoupler. The CdS photocell was used as (**a**) a single resistor, (**b**) a resistor $R_{X}$ of the bioimpedance model, or (**c**) $R_{\mathrm{PX}}$ of its model.

Furthermore, a CdS photocoupler (LCR0203, Nanyang Senba Optical and Electronic, Shenzhen, China) was incorporated into the oscillator circuit as a part of ${\dot{Z}}_{X}$, and the temporal change in $R_{X}$ and $R_{\mathrm{PX}}$ could be measured using the proposed method. CdS photocells can be regarded as simple resistors. Moreover, increasing the current $I_{\mathrm{Ph}}$ flowing through a light-emitting diode (LED) built in the CdS photocoupler decreases the resistance value of the CdS photocell. Therefore, the resistance value can be changed over time by changing the current $I_{\mathrm{Ph}}$ of the LED. This current was controlled via a constant current circuit using an operational amplifier (NJU7032D, NJR, Tokyo, Japan) and an N-channel field-effect transistor (2N7000, ON Semiconductor, Phoenix, USA), as illustrated in Figure 3. In this circuit, the current $I_{\mathrm{Ph}}$ can be controlled in the range 0.6–10 mA by adjusting the voltage $V_{C}$ between 0.12 and 2 V. The capacitive coupling $C_{\mathrm{SX}}$ was set to 9.76 nF. First, it was measured when only the photocell was connected as a resistor $R_{X}$ via $C_{\mathrm{SX}}$ (Figure 3a). In this case, the counter IC and analog switch IC of the mode-switching nonsinusoidal oscillator were not used because the parameters could be estimated sufficiently accurately with a single mode of $R_{F}$. Only the $R_{F1}$ of 8.2 kΩ was connected as $R_{F}$. Subsequently, the temporal change in the impedance spectrum was determined from the oscillation waveform of the oscillator using the bioimpedance model and $C_{\mathrm{SX}}$ (Figure 3b,c). $R_{F3}$ is short circuited so that the oscillation frequency is switched in two modes to increase the temporal resolution. $R_{F1}$ and $R_{F2}$ were 3.3 and 1.8 kΩ, respectively.

#### 2.2.4. Data Acquisition and DFT Analysis

Time waveform data of voltages were collected using a digital oscilloscope (PicoScope 5444D, Pico Technology, Cambridgeshire, UK). $v_{1}\left(t\right)$ and $v_{2}\left(t\right)$ in Figure 1 were measured, and time waveform data for $v_{12}\left(t\right)$ were obtained from the difference between them. Thereafter, DFT was performed for the obtained waveform data. When using the mode-switching function, the oscillation frequency is switched in four or two modes every two cycles. DFT was performed individually for the waveform of the second half cycle of each oscillation frequency, and the merged spectral data for the set of oscillation frequencies (one segment) were obtained. DFT was performed for every cycle of the oscillation waveform when mode switching was not used. A rectangular window was used for the window function of DFT. Furthermore, the impedance spectra for 10 segments were averaged, except for experiments using a CdS photocoupler. The real and imaginary parts of ${\dot{Z}}_{X}$ were calculated from the obtained DFT data, and $R_{X},\;R_{\mathrm{PX}},C_{\mathrm{PX}},\;\mathrm{and}\;C_{\mathrm{SX}}$ were estimated via the least squares method using Equations (7) and (8) or (9) and (10). In the estimation using Equations (9) and (10), first, $R_{X},\;R_{\mathrm{PX}},\;\mathrm{and}\;C_{\mathrm{PX}}$ were estimated using Equation (9) from the DFT data of the real part of ${\dot{Z}}_{X}$. The estimated $R_{X},\;R_{\mathrm{PX}},\;\mathrm{and}\;C_{\mathrm{PX}}$ were then substituted into Equation (10), and $C_{\mathrm{SX}}$ was estimated from the DFT data of the imaginary part of ${\dot{Z}}_{X}$. In the circuit parameter estimation, odd-order harmonic components up to the 19th order obtained through DFT are used. However, because the frequency characteristics of the PP film capacitor and parasitic capacitance of the circuit board affect the estimation result in high-frequency ranges, harmonic components greater than 500 kHz when $C_{\mathrm{PX}}$ was less than 2.0 nF and harmonic components greater than 350 kHz when $C_{\mathrm{PX}}$ was 2.0 nF or higher were not used for estimation.

## 3. Results and Discussion

### 3.1. CIS of Parallel RC Circuits and Bioimpedance Models

**Figure 4:**
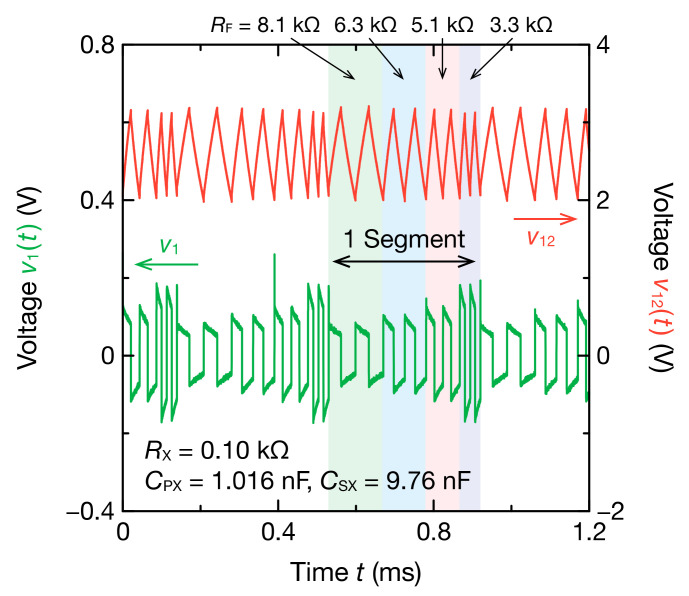
Four-mode oscillation waveforms of $v_{1}\left(t\right)$ and $v_{12}\left(t\right)$ obtained through the mode-switching nonsinusoidal oscillator. $R_{X}$, $R_{\mathrm{PX}}$, $C_{\mathrm{PX}}$, and $C_{\mathrm{SX}}$ were set to 0.10 k, 0 Ω, 1.016 nF, and 9.76 nF, respectively. The oscillation frequency was switched every two cycles of each $R_{F}$. The DFT analysis was performed on the oscillation waveforms of the second half of two cycles.

Figure 4 illustrates oscillation waveforms when $\;R_{X}$ = 0.10 kΩ, $R_{\mathrm{PX}}$ = 0 Ω, $C_{\mathrm{PX}}\;$= 1.016 nF, and $C_{\mathrm{SX}}\;$= 9.76 nF are connected as ${\dot{Z}}_{x}$ in the mode-switching nonsinusoidal oscillator. $R_{F}$ switched every two cycles, and the oscillation frequency changed in four modes. Oscillating frequencies when $R_{F}$ was 8.1, 6.3, 5.1, and 3.3 kΩ were 14.0, 18.2, 23.2, and 38.4 kHz, respectively. The smaller values than the values estimated using Equation (1) may be because the actual threshold voltages of the Schmitt trigger IC are different from the typical values in the datasheet specification. The spike noise observed at 0.4 ms is due to the switching noise of the Schmitt trigger inverter. However, the spike noise has no effect on the estimation of the impedance spectrum because we used only the waveform of the second half cycle for DFT.

**Figure 5:**
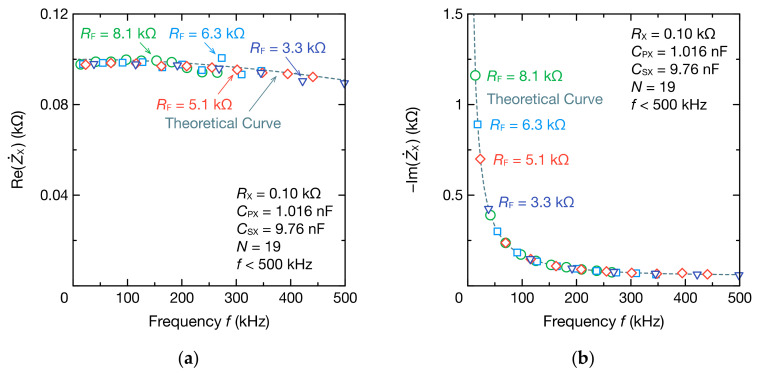
Frequency spectra of the (**a**) real part ReZ˙X and (**b**) imaginary part ImZ˙X of the impedance ${\dot{Z}}_{X}$ comprising the resistance $R_{X}$ of 0.10 kΩ, resistance $R_{\mathrm{PX}}$ of 0 Ω, capacitance $C_{\mathrm{PX}}$ of 1.016 nF (parallel RC circuit), and capacitance $C_{\mathrm{PX}}$ of 9.76 nF. The dashed lines represent the theoretical curves calculated from Equations (9) and (10).

**Figure 6:**
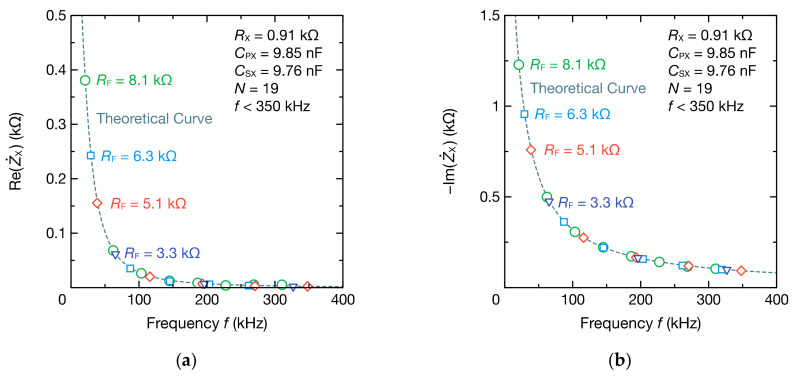
Frequency spectra of the (**a**) real part ReZ˙X and (**b**) imaginary part ImZ˙X of the impedance ${\dot{Z}}_{X}$ comprising the resistance $R_{X}$ of 0.91 kΩ, resistance $R_{\mathrm{PX}}$ of 0 Ω, capacitance $C_{\mathrm{PX}}$ of 9.85 nF (parallel RC circuit), and capacitance $C_{\mathrm{SX}}$ of 9.76 nF. The dashed lines represent the theoretical curves calculated from Equations (9) and (10).

Figure 5 shows the spectra of ${\dot{Z}}_{X}$ calculated using DFT analysis. By applying DFT to the oscillation waveform for each $R_{F}$, four modes of frequency spectra were obtained. These spectra agreed well with the theoretical values calculated from Equations (9) and (10) with $R_{\mathrm{PX}}$ = 0 Ω. Even when the resistance and capacitance were large, such as $R_{X}\;$= 0.91 kΩ and $C_{\mathrm{PX}}\;$= 9.85 nF, impedance spectra agreed well with the theoretical values (Figure 6).

**Figure 7:**
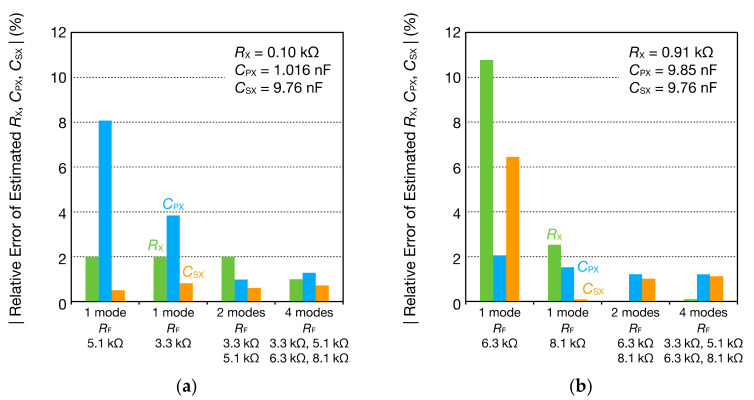
Absolute relative estimation errors of the circuit parameters of parallel RC circuits: (**a**) $R_{X}$ = 0.10 kΩ, $C_{\mathrm{PX}}$ = 1.016 nF, $\mathrm{and}\;C_{\mathrm{SX}}$ = 9.76 nF; (**b**) $R_{X}$ = 0.91 kΩ, $C_{\mathrm{PX}}$ = 9.85 nF, and $C_{\mathrm{SX}}$ = 9.76 nF.

Further, the values of $R_{X},\;\;C_{\mathrm{PX}},\;\mathrm{and}\;C_{\mathrm{SX}}$ were estimated from the impedance spectrum. Figure 7 illustrates the estimation values of $R_{X},\;C_{\mathrm{PX}},\;\mathrm{and}\;C_{\mathrm{SX}}$. To verify the usefulness of the mode-switching function, the estimation was performed using the spectrum obtained from the four combinations of $R_{F}$ and the spectrum obtained from one mode and two modes of $R_{F}$. The estimation error sometimes exceeded 2% when only DFT data obtained from one mode of $R_{F}$ were used; however, the estimation accuracy improved when two modes of $R_{F}$ were used. Moreover, each circuit parameter could be estimated with high accuracy, regardless of the value of $R_{X}\;\mathrm{and}\;C_{\mathrm{PX}}$, using the four modes of $R_{F}$. This demonstrates that the superposition of the impedance spectra through the mode-switching function improves the estimation accuracy of the circuit parameters.

**Figure 8:**
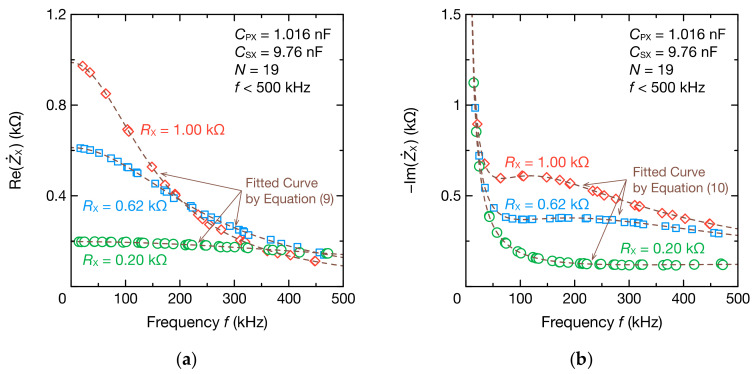
Frequency spectra of the (**a**) real part ReZ˙X and (**b**) imaginary part ImZ˙X of the impedance ${\dot{Z}}_{X}$ comprising the parallel RC circuits for $C_{\mathrm{PX}}=$ 1.016 nF and coupling capacitance $C_{\mathrm{SX}}=$ 9.76 nF. The DFT data were fitted to Equations (9) and (10) to calculate $R_{X}$, $C_{\mathrm{PX}}$, and $C_{\mathrm{SX}}$ (dashed lines).

Subsequently, with $C_{\mathrm{PX}}$ = 1.016 nF and $C_{\mathrm{SX}}$ = 9.76 nF, we measured the impedance spectrum by changing $R_{X}$ from 0.1 to 1 kΩ. Figure 8 shows some of the impedance spectra obtained. The impedance spectra according to the values of $R_{X}$ were obtained, and the fitting curves obtained from Equations (9) and (10) using the least squares method agreed well with the DFT data.

**Figure 9:**
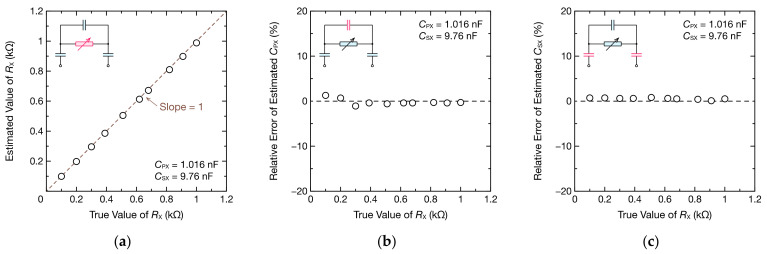
$R_{X}$, $C_{\mathrm{PX}},$ and $C_{\mathrm{SX}}$ of the parallel RC circuits estimated using oscillation waveforms and DFT: (**a**) the estimated $R_{X}$, (**b**) relative error of the estimated $C_{\mathrm{PX}}$, and (**c**) relative error of the estimated $C_{\mathrm{SX}}$. The insets are the circuit model used for fitting, and the estimated parameters are highlighted in pink.

When the circuit parameters were estimated, the estimation error of $R_{X}$ was within 12 Ω, and $R_{X}$ could be accurately estimated from 0.1 to 1 kΩ (Figure 9a). Furthermore, the errors of $C_{\mathrm{PX}}$ and $C_{\mathrm{SX}}$ were within 1.28% and 0.82%, respectively, which are sufficiently small errors (Figure 9b,c). These results indicate that the circuit parameters of the RC parallel circuit can be estimated with high accuracy through capacitive coupling.

**Figure 10:**
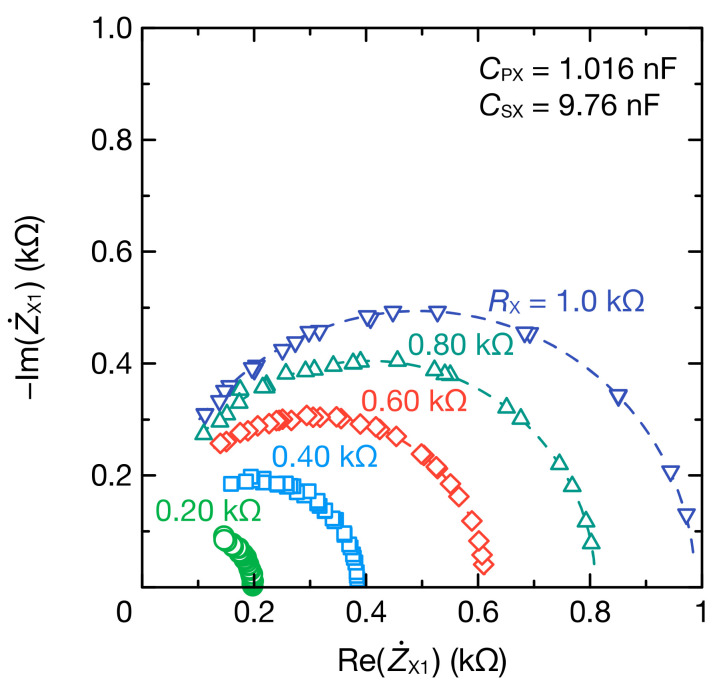
Cole–Cole plot of ${\dot{Z}}_{X1}$ for the parallel RC circuits with $C_{\mathrm{PX}}=$1.016 nF and $C_{\mathrm{SX}}=$9.76 nF. The dashed lines represent the fitting curves obtained from Equations (9) and (10).

The impedance characteristics of a measured object are frequently discussed using Cole–Cole plots. The proposed method can obtain a wide frequency range spectrum using multiple values of $R_{F}$, and the Cole–Cole plots, as illustrated in Figure 10, can be easily obtained. The Cole–Cole plot of the impedance ${\dot{Z}}_{X1}$ excluding capacitive coupling showed semicircles, and the radius of the semicircle increased with resistance $R_{X}$.

**Figure 11:**
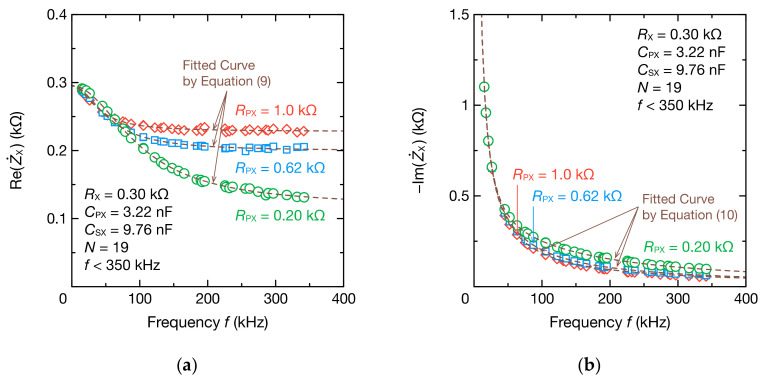
Frequency spectra of the (**a**) real part ReZ˙X and (**b**) imaginary part ImZ˙X of the impedance ${\dot{Z}}_{X}$ comprising the bioimpedance models for $R_{X}=$0.30 kΩ, $C_{\mathrm{PX}}=$3.22 nF, and coupling capacitance $C_{\mathrm{SX}}=$9.76 nF. DFT data were fitted to Equations (9) and (10) to determine $R_{X}$, $R_{\mathrm{PX}}$, $C_{\mathrm{PX}},$ and $C_{\mathrm{SX}}$ (dashed lines).

**Figure 12:**
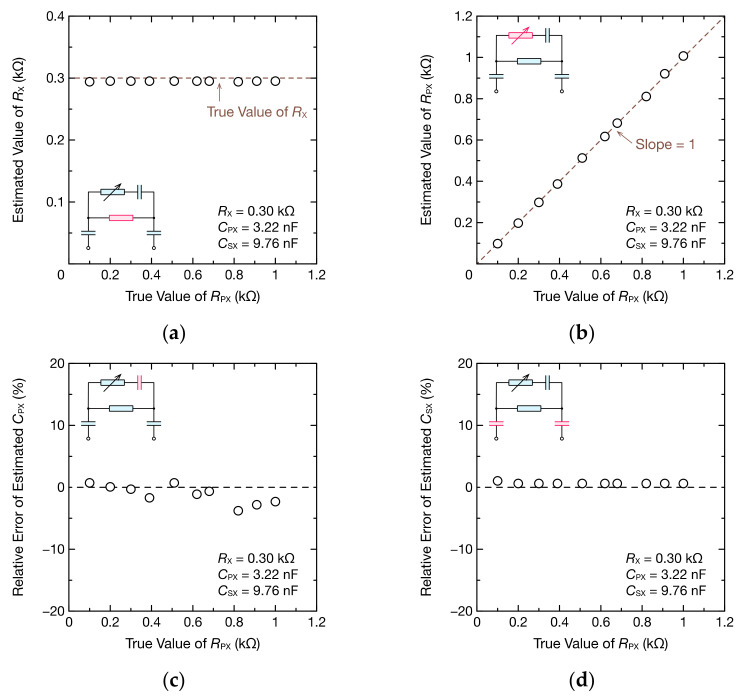
$R_{X}$, $C_{\mathrm{PX}}$, and $C_{\mathrm{SX}}$ of the bioimpedance models estimated using oscillation waveforms and DFT: (**a**) the estimated $R_{X}$, (**b**) the estimated $R_{\mathrm{PX}}$, (**c**) relative error of the estimated $C_{\mathrm{PX}}$, and (**d**) relative error of the estimated $C_{\mathrm{SX}}$. The insets are the circuit models used for fitting, and the estimated parameters are highlighted in pink.

Moreover, we investigated whether the circuit parameters of the bioimpedance model could be estimated. Figure 11 shows the impedance spectrum of the bioimpedance model when $R_{\mathrm{PX}}$ was 0.20, 0.62, or 1.0 kΩ. Despite the small change in the real part compared with the RC parallel circuit due to the existence of $R_{\mathrm{PX}}$, the fitting results using Equation (9) agreed well with the DFT data. The imaginary part could also be fitted accurately using Equation (10). Figure 12 shows the estimation results of $R_{X},\;R_{\mathrm{PX}},\;C_{\mathrm{PX}},\;\mathrm{and}\;C_{\mathrm{SX}}$. The estimation errors of $R_{X}$ and $R_{\mathrm{PX}}$ were within 6 and 11 Ω, respectively, which are sufficiently small estimation errors. The estimation errors of $C_{\mathrm{PX}}\;\mathrm{and}\;C_{\mathrm{SX}}$ were also within 3.79% and 1.03%, respectively.

**Figure 13:**
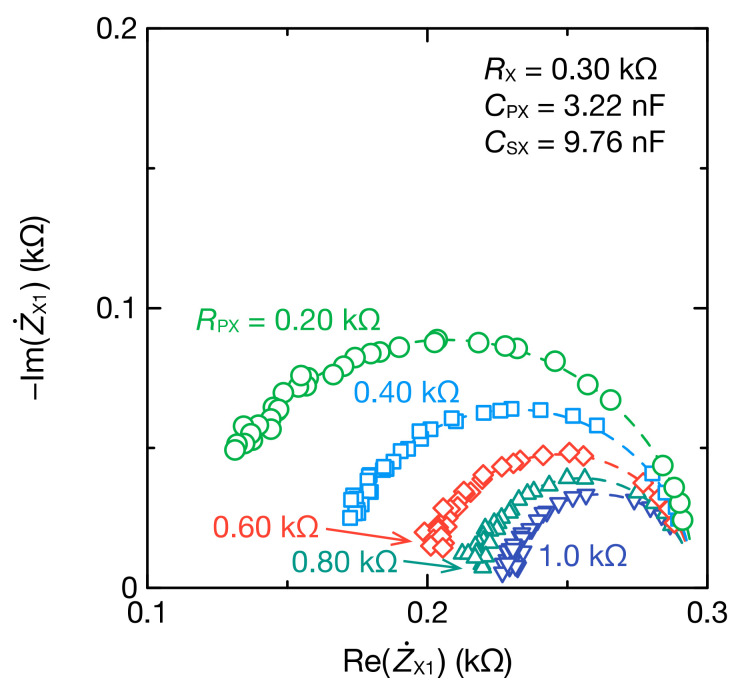
Cole–Cole plot of ${\dot{Z}}_{X1}$ for the bioimpedance models with $C_{\mathrm{PX}}=$3.22 nF and $C_{\mathrm{SX}}=$9.76 nF. The dashed lines represent the fitting curves obtained from Equations (9) and (10).

Figure 13 illustrates the Cole–Cole plots of the bioimpedance models excluding capacitive coupling. The Cole–Cole plot of ${\dot{Z}}_{X1}$ showed semicircles expected from Equations (9) and (10), and the radius of the semicircle decreased with resistance $R_{\mathrm{PX}}$.

These results demonstrate that the circuit parameters can be estimated using the proposed method, even for complex equivalent circuit models such as bioimpedance. In this study, $R_{F}$ over 1 kΩ was not estimated because we designed the mode-switching oscillator to apply to biological tissue. Our proposed oscillator generates a stable oscillation waveform if the feedback resistance is sufficiently larger than the magnitudes of ${\dot{Z}}_{X}$ and ${\dot{Z}}_{A}$. Therefore, the range of load that our circuit can carry depends on the feedback resistance. Resistance of less than several kΩ can be estimated when the feedback resistance is about 10 kΩ. If the feedback resistance is higher, we will be able to estimate higher resistance of about 10 kΩ.

### 3.2. Application of CIS to Biological Tissues

**Figure 14:**
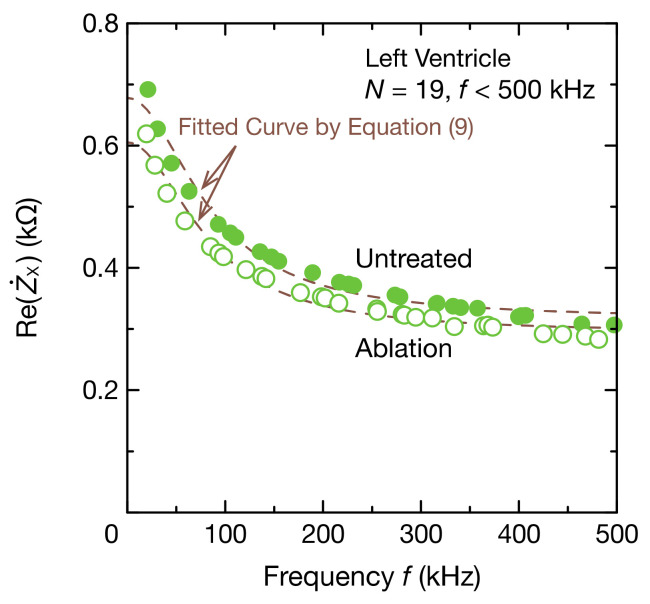
Frequency spectrum of the real part ReZ˙X of the impedance ${\dot{Z}}_{X}$ of the porcine myocardium and coupling capacitance. DFT data were fitted to Equation (9) to determine $R_{X}$, $R_{\mathrm{PX}}$, and $C_{\mathrm{PX}}$ (dashed lines).

An example of the application of impedance measurement in the biomedical field is the monitoring of cauterization in catheter ablation. We, therefore, used CIS to measure the local change in impedance when porcine myocardium was coagulated. Stable oscillations with four modes of oscillating frequencies were obtained when the electrodes in Figure 2b and the surface of the porcine myocardium were in contact. Figure 14 shows the spectrum of the real part of the impedance ${\dot{Z}}_{X}$ calculated using DFT from the obtained waveform. The equivalent circuit of biological tissues, such as porcine myocardium, is approximated using a bioimpedance model. Therefore, the real part of the impedance decreased with increasing frequency as the capacitance of cell membranes decreases. Moreover, because the measurement area included regions other than the ablation site, the impedance was larger than the local impedance (approximately 0.10 kΩ) measured through a commercial catheter ablation system. When electrocautery was performed, the impedance of the porcine myocardium decreased, regardless of the frequency range. Because cell membranes at the ablation site have high ion permeability, this decrease in impedance indicates that a part of the myocardium was degenerated through coagulation. The DFT data partially agree with the fitting curve produced by Equation (9), but the DFT data tended to be larger than the fitted data in low-frequency ranges. Furthermore, when $C_{\mathrm{SX}}$ was estimated using Equation (10) from the DFT data of the imaginary part of the impedance, the value before ablation was 7.98 nF and the value after ablation was 8.29 nF, both of which are low values compared to the capacitance of the PP film capacitor used, which was 9.76 nF. This suggests that resistance and capacitance components also exist between the surface of the porcine myocardium and electrodes. Therefore, to estimate circuit parameters with high accuracy, applying an equivalent circuit model is necessary considering the impedance of the interface of the myocardium and electrodes.

**Figure 15:**
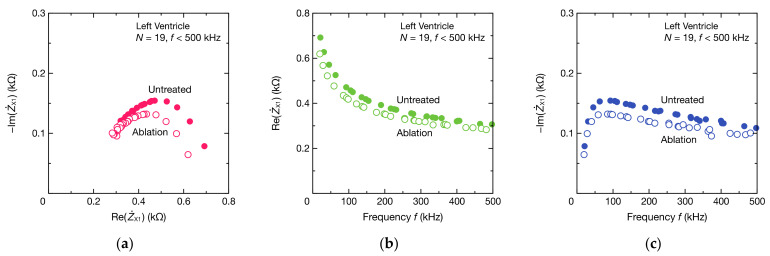
Frequency spectra of the impedance ${\dot{Z}}_{X1}$ of the porcine myocardium: (**a**) the Cole–Cole plot, (**b**) the real part ReZ˙X1, and (**c**) the imaginary part ImZ˙X1. ${\dot{Z}}_{X1}$ represents the impedance obtained by subtracting the impedance of the estimated $C_{\mathrm{SX}}$ from ${\dot{Z}}_{X}$. ReZ˙X1 equals ReZ˙X in Figure 14.

Figure 15 shows the frequency spectra of the porcine myocardium. ${\dot{Z}}_{X1}$ in Figure 15 represents the impedance of the porcine myocardium only, excluding the impedance of the capacitive coupling $C_{\mathrm{SX}}$ from ${\dot{Z}}_{X}$. Here, $C_{\mathrm{SX}}$ uses a value estimated through fitting, not the capacitance value of the PP film capacitor. The Cole–Cole plot of the porcine myocardium formed a semicircle, as expected from the bioimpedance model, but the semicircle was distorted (Figure 15a). This may be because the impedance between the surface of the porcine myocardium and electrodes was not considered or because the impedance of the myocardial tissue was not uniform. When the myocardium was ablated, the magnitude of the imaginary part of the impedance also decreased, similar to the real part (Figure 15b,c), as did the radius of the semicircle in the Cole–Cole plot.

**Figure 16:**
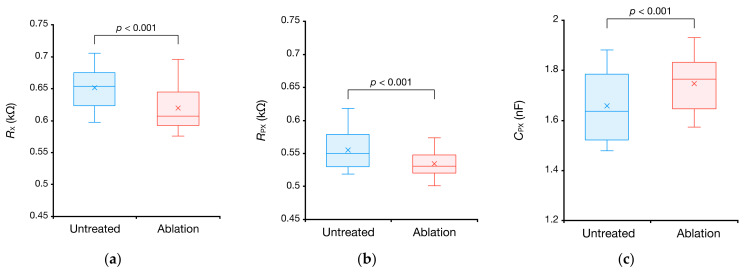
Comparison of equivalent circuit parameters between the untreated and ablated left ventricles of porcine hearts: (**a**) $R_{X}$, (**b**) $R_{\mathrm{PX}}$, and (**c**) $C_{\mathrm{PX}}$. The sample size is 12.

Furthermore, impedance was measured at four locations using CIS for each of the three porcine myocardium samples. Figure 16 shows the result of estimating $R_{X}$, $R_{\mathrm{PX}}$, and $C_{\mathrm{PX}}$ from the determined impedance spectra. Ablation decreased the resistive components $R_{X}$ and $R_{\mathrm{PX}}$ and increased the capacitive component $C_{\mathrm{PX}}$ in all 12 samples. A paired t-test on the obtained samples also showed significant differences at a significance level of 0.1%, indicating that CIS can measure changes in impedance associated with myocardial tissue degeneration.

### 3.3. Continuous Impedance Measurement Using CIS

**Figure 17:**
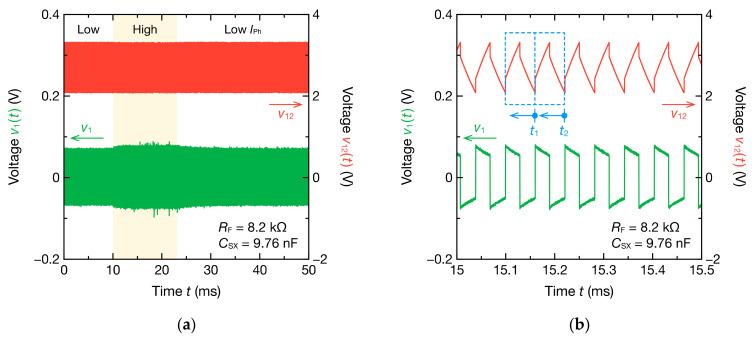
Single-mode oscillation waveforms of $v_{12}\left(t\right)$ and $v_{1}\left(t\right)$ for the nonsinusoidal oscillator incorporating the CdS photocell (Figure 3a): (**a**) full waveform and (**b**) enlarged waveform. The photocurrent $I_{\mathrm{Ph}}$ of the photocoupler is changed at 10 and 23 ms. The high and low levels of $I_{\mathrm{Ph}}$ are 2.0 and 0.6 mA, respectively. Circuit parameters are estimated for each cycle (blue dashed line). Representative points of time in each cycle are the ends of the cycles such as $t_{1}$ and $t_{2}$.

Figure 17 shows the oscillation waveform when a CdS photocoupler and a 9.76 nF PP film capacitor are connected in series as ${\dot{Z}}_{X}$ (Figure 3a). Measurement was started with a current $I_{\mathrm{Ph}}$ of 0.6 mA flowing through the LED, and the current $I_{\mathrm{Ph}}$ was increased instantaneously to 2.0 mA at 10 ms after the start of the measurement. Thereafter, the current $I_{\mathrm{Ph}}$ was returned to 0.6 mA again at 23 ms after the start of the measurement. Figure 17a shows that the switching of the current $I_{\mathrm{Ph}}$ changed the peak-to-peak value of the voltage $v_{1}\left(t\right)$, indicating that the resistance of the CdS cell changed. The peak-to-peak value $V_{\mathrm{PP}}$ of the voltage $v_{1}\left(t\right)$ is expressed by the following equation:

(11) $$V_{\mathrm{PP}}=\frac{R_{A}}{R_{F}}\left(V_{P}-V_{N}\right)+\frac{R_{A}}{R_{F}}\left[\frac{2R_{F}}{R_{A}+R_{X}+R_{F}}-1\right]\left(V_{H}-V_{L}\right).$$

As shown in this equation, $V_{\mathrm{PP}}$ increases with decreasing resistance $R_{X}$. Figure 17b indicates that a stable oscillation was obtained.

**Figure 18:**
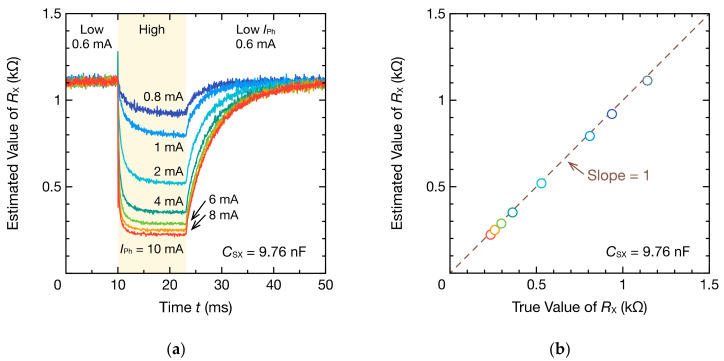
Resistance $R_{X}$ of the photocell estimated using oscillation waveforms in Figure 17a and DFT: (**a**) time–resistance curves and (**b**) saturation values at a high $I_{\mathrm{Ph}}$ period. Time of the time–resistance curves corresponds to the representative points of time in Figure 17b.

Continuous frequency spectra were obtained through DFT from the waveforms of the respective cycles of Figure 17a, and the resistance $R_{X}$ of the CdS photocell could be estimated by applying Equation (7). The temporal waveform of $R_{X}$ obtained by estimation is presented in Figure 18a. When $I_{\mathrm{Ph}}$ was increased instantaneously, $R_{X}$ gradually decreased and then saturated at approximately 10 ms after the current increased. Because the response time of the CdS photocell used is relatively short, approximately 2.5 ms, this change in $R_{X}$ reflects the optical response of the CdS photocell. The increase from 24 ms indicates that the resistance of the photocell increased with the reduction in $I_{\mathrm{Ph}}$. Generally, the recovery time of the photocell is slower than the response time. Therefore, the gradual increase in the resistance is a reasonable response. Figure 18b compares the saturated and true values of $R_{X}$ at various $I_{\mathrm{Ph}}$ values. The error between the estimated and true $R_{X}$ values was 29 Ω at the maximum, and thus $R_{X}$ could be estimated accurately.

**Figure 19:**
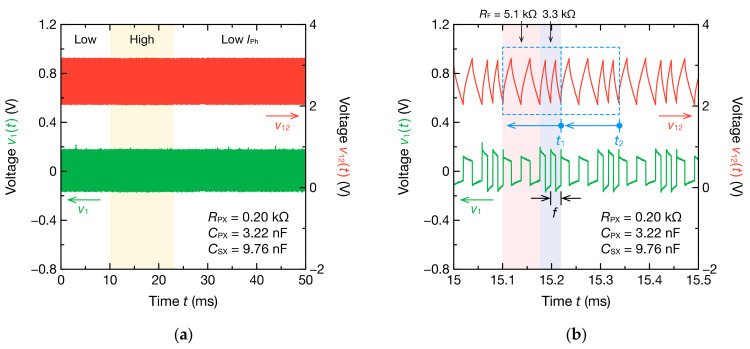
Two-mode oscillation waveforms of $v_{12}\left(t\right)$ and $v_{1}\left(t\right)$ for the nonsinusoidal oscillator incorporating the CdS photocell and circuit elements of the bioimpedance model (Figure 3b): (**a**) full waveform and (**b**) enlarged waveform. The photocurrent $I_{\mathrm{Ph}}$ of the photocoupler is changed at 10 and 23 ms. The high and low levels of $I_{\mathrm{Ph}}$ are 8.0 and 4.0 mA, respectively. Circuit parameters are estimated for each cycle (blue dashed line). Representative points of time in each cycle are the ends of the cycles such as $t_{1}$ and $t_{2}$. The frequency *f* is the oscillation frequency obtained from the second half cycle of the oscillation waveform at $R_{F}$ = 3.3 kΩ.

Moreover, a CdS photocell was incorporated as $R_{X}$ or $R_{\mathrm{PX}}$ in the bioimpedance model (Figure 3b,c), and the resistance of the CdS photocell was changed over time. Figure 19 shows the oscillation waveform when the resistance of the CdS photocell was changed temporally as $R_{X}$. Measurement began with a current $I_{\mathrm{Ph}}$ of 4.0 mA flowing through the LED, and the current $I_{\mathrm{Ph}}$ was increased instantaneously to 8.0 mA at 10 ms after the measurement began. Thereafter, $I_{\mathrm{Ph}}$ was returned to 4.0 mA again at 23 ms after the start of the measurement. $R_{F}$ was 3.3 or 5.1 kΩ, and mode switching was performed in two modes.

**Figure 20:**
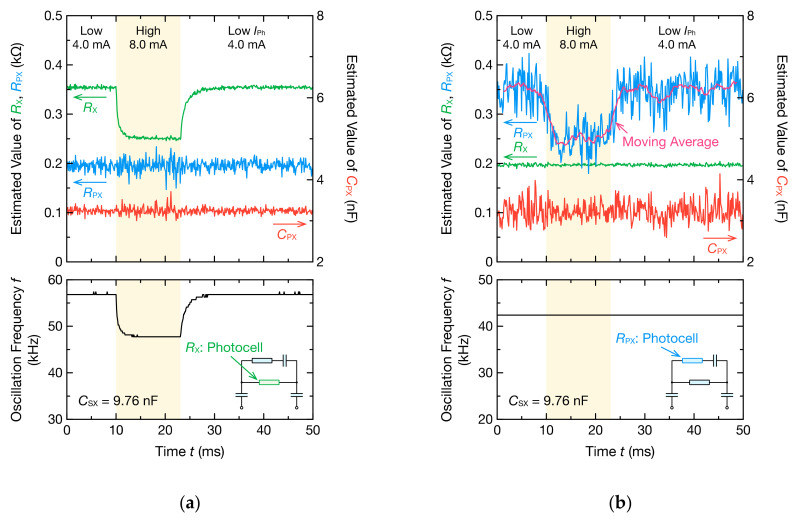
Time–resistance, time–capacitance, and time–frequency curves of the resistance of the photocell and circuit elements of the bioimpedance model estimated using oscillation waveforms and DFT. The photocell was used as (**a**) $R_{X}$ or (**b**) $R_{\mathrm{PX}}$. The other resistance is 0.20 kΩ; the capacitance $C_{\mathrm{PX}}$ is 3.22 nF; and the capacitance $C_{\mathrm{SX}}$ is 9.76 nF. The times of these curves correspond to the representative points of time in Figure 19b. The insets are photocell-incorporated circuits.

A stable oscillation was obtained, as illustrated in Figure 19b, and the oscillation frequency switched in two modes. A $V_{\mathrm{PP}}$ change was not observed because the change in $I_{\mathrm{Ph}}$ was smaller than that shown in Figure 17a. Although Equation (11) is not strictly valid when $C_{\mathrm{PX}}$ is included in ${\dot{Z}}_{X}$, $V_{\mathrm{PP}}$ depends on $R_{X}$ connected in parallel with $R_{\mathrm{PX}}$ and $C_{\mathrm{PX}}$. However, because the resistance change due to the switching of $I_{\mathrm{Ph}}$ was only about 0.1 kΩ, the $V_{\mathrm{PP}}$ change was negligibly small. Further, when the temporal change in $R_{X}$, $R_{\mathrm{PX}}$, and $C_{\mathrm{PX}}$ was estimated through the DFT analysis of the oscillation waveform, only $R_{X}$ changed according to the change in the CdS photocell resistance (Figure 20a). Furthermore, even when $R_{\mathrm{PX}}$ and $C_{\mathrm{PX}}$ are connected in parallel with $R_{X}$, the oscillation frequency changes with $R_{X}$ of the measured object and is not significantly affected by electrical noise. Therefore, when the equivalent circuit model of the measured object is unknown or the signal-to-noise ratio in the circuit is low, the oscillation frequency can be used for monitoring the circuit parameters. However, the change in $R_{\mathrm{PX}}$ was not reflected in the oscillation frequency when the CdS photocell was incorporated as $R_{\mathrm{PX}}$ (Figure 20b). The oscillation frequency also depends on the impedance ${\dot{Z}}_{X}$ including $R_{\mathrm{PX}}$. However, $R_{\mathrm{PX}}$ is connected in series with $C_{\mathrm{PX}}$, and $C_{\mathrm{PX}}$ has a relatively large impedance at the oscillation frequency. Therefore, the change in $R_{\mathrm{PX}}$ does not significantly contribute to the changes in ${\dot{Z}}_{X}$, which leads to no change in the oscillation frequency.

DFT analysis indicated that only $R_{\mathrm{PX}}$ changed, but the dispersion of the data was particularly large for $R_{\mathrm{PX}}$. The estimation accuracy of $R_{\mathrm{PX}}$ depends on the measurement accuracy of the impedance in high-frequency ranges, but the spectra of high-frequency ranges are susceptible to noise because the signal of the harmonic component is smaller than the fundamental frequency component. Therefore, the estimation accuracy of $R_{\mathrm{PX}}$ may be poor. However, because one segment of the oscillation waveform is sufficiently short and the time resolution is high, the estimation accuracy can be improved by considering the moving average. The above results indicate that the proposed method can be used to continuously monitor the temporal variation in impedance.

## 4. Conclusions

We fabricated a mode-switching nonsinusoidal oscillator to accurately estimate the circuit parameters of impedance, including multiple resistances and capacitances, using CIS. The oscillation waveform of the fabricated circuit had multiple oscillation frequencies caused by the periodic switching of the feedback resistor. This enabled us to obtain a broader frequency spectrum range with a higher resolution than a single oscillation frequency through DFT analysis. Further, the circuit parameters of complicated circuit models such as RC parallel circuits and bioimpedance models could also be estimated accurately from the oscillation waveforms of this circuit.

We applied CIS using the mode-switching nonsinusoidal oscillator to impedance measurement of porcine myocardium. Consequently, the changes in the local impedance spectrum caused by the coagulation of the porcine myocardium could be measured. Moreover, the resistive components decreased and the capacitive components increased during electrocautery by fitting the impedance spectra with the bioimpedance model. By measuring and analyzing the continuous oscillation waveform when a CdS photocell was incorporated into the mode-switching nonsinusoidal oscillator, measuring the changes in impedance over time was possible.

These findings demonstrate that temporal changes in the impedance of biological tissues can be monitored using our proposed method. However, because this study excluded the impedance measurements of biological tissues through capacitive coupling using an insulator, future studies should aim to apply CIS to biological tissues covered with an insulator, such as the human body with clothing.

## Figures and Tables

**Table 1:** Four different modes of the feedback resistance $R_{F}$.

Mode	$Q_{1}$	$Q_{0}$	SW1	SW2	$R_{F}$
1	0	0	OFF	OFF	$R_{F1}+R_{F2}+R_{F3}$
2	0	1	OFF	ON	$R_{F1}+R_{F3}$
3	1	0	ON	OFF	$R_{F1}+R_{F2}$
4	1	1	ON	ON	$R_{F1}$

0 and 1 indicate a low level and a high level of a square wave, respectively.

**Table 2:** Two sets of the oscillation frequencies obtained by combining $R_{F1}$, $R_{F2}$, and $R_{F3}$.

Set	$R_{F1}$ (kΩ)	$R_{F2}$ (kΩ)	$R_{F3}$ (kΩ)	4 Modes of $R_{F}$ (kΩ)	Estimated Oscillation Frequencies (kHz)
1	3.3	1.8	3	8.1, 6.3, 5.1, 3.3	16.9, 22.3, 28.4, 47.9
2	5.1	1	2	8.1, 7.1, 6.1, 5.1	16.9, 19.5, 23.1, 28.4

The oscillation frequencies were estimated using Equations (1)–(3) with $R_{X}=$ 0 Ω. The actual oscillation frequencies depend on ${\dot{Z}}_{X}$.

## Data Availability

The data that support the findings of this study are available from the corresponding author, A.U., upon reasonable request.
